# SOCIAL AND ECONOMIC SUPPORT FOR PEOPLE LIVING WITH DEMENTIA IN MEXICO

**DOI:** 10.1093/geroni/igae098.1152

**Published:** 2024-12-31

**Authors:** Mariana Lopez-Ortega, Rosa Garcia-Chanes

**Affiliations:** Instituto Nacional de Geriatria, Mexico City, Distrito Federal, Mexico; Instituto Nacional de Geriatria, Mexico City, Distrito Federal, Mexico

## Abstract

Dementia is one of the most common causes of disability and dependence in the world and is a frequent reason that older people require supportive living situations. In Mexico, Alzheimer’s and other dementias represent the fifth main cause of DALYS (disability-adjusted life-years) and it is estimated that there were approximately 1 626 000 persons living with dementia. However, the health system is not prepared to adequately provide diagnosis, post-diagnosis care as well as care support at home for people living with dementia and their family caregivers. In addition, economic conditions of a large number of older persons is precarious given a life of informal work with no benefits or access to a contributory pension in old age and whose sole income is a small non-contributory pension and relying on children or other close relatives to provide financial and in-kind support, as well as all other health and personal care. The aim of this study is to characterise the support received by a sample of older adults in Mexico. We use the Mexican Health and Aging Study 2016 wave which for the first time included the Mex-Cog Wave 1 a detailed cognitive assessment. This allows for a detailed characterisation of support received by people living with dementia and to compare it with the support available and received by older adults with no impairment. The results allow for specific recommendations for further support programs and activities that focus on areas of need and currently not covered either by public or family supports.

